# High bacterial and viral load in the upper respiratory tract of children in the Democratic Republic of the Congo

**DOI:** 10.1371/journal.pone.0240922

**Published:** 2020-10-29

**Authors:** Archippe Muhandule Birindwa, Lucia Gonzales-Siles, Rickard Nordén, Shadi Geravandi, Jeanière Tumusifu Manegabe, Lambert Morisho, Stay Saili Mushobekwa, Rune Andersson, Susann Skovbjerg

**Affiliations:** 1 Department of Infectious Diseases, Institute of Biomedicine, Sahlgrenska Academy, University of Gothenburg, Gothenburg, Sweden; 2 Panzi Hospital, Bukavu, Democratic Republic of the Congo; 3 Université Evangélique en Afrique, Bukavu, Democratic Republic of the Congo; 4 Institut Superieur Technique Medical, Uvira, Democratic Republic of the Congo; 5 Department of Clinical Microbiology, Sahlgrenska University Hospital, Gothenburg, Region Västra Götaland, Sweden; Defense Threat Reduction Agency, UNITED STATES

## Abstract

**Background:**

Respiratory pathogens including *Streptococcus pneumoniae* and *Haemophilus influenzae*, are implicated in the pathogenicity of acute lower respiratory infection (ALRI). These are also commonly found in both healthy and sick children. In this study, we describe the first data on the most frequent bacteria and viruses detected in the nasopharynx of children from the general population in the Eastern DR Congo.

**Methods:**

From January 2014 to June 2015, nasopharyngeal samples from 375 children aged from 2 to 60 months attending health centres for immunisation or growth monitoring were included in the study. Multiplex real-time PCR assays were used for detection of 15 different viruses and 5 bacterial species and for determination of pneumococcal serotypes/serogroups in the nasopharyngeal secretions.

**Results:**

High levels of S. *pneumonia*e were detected in 77% of cases, and *H*. *influenzae* in 51%. Rhinovirus and enterovirus were the most commonly found viruses, while respiratory syncytial virus (RSV) was rare (1%). Co-occurrence of both bacteria and viruses at high levels was detected in 33% of the children. The pneumococcal load was higher in those children who lived in a dwelling with an indoor kitchen area with an open fire, i.e. a kitchen with an open fire for cooking located inside the dwelling with the resultant smoke passing to the living room and/or bedrooms; this was also higher in children from rural areas as compared to children from urban areas or children not living in a dwelling with an indoor kitchen area with an open fire/not living in this type of dwelling. Immunization with 2–3 doses of PCV13 was associated with lower rates of pneumococcal detection. Half of the identified serotypes were non-PCV13 serotypes. The most common non-PCV13 serotypes/serogroups were 15BC, 10A, and 12F, while 5, 6, and 19F were the most prevalent PCV13 serotypes/serogroups.

**Conclusions:**

The burden of respiratory pathogens including S. *pneumonia*e in Congolese children was high but relatively few children had RSV. Non-PCV13 serotypes/serogroups became predominant soon after PCV13 was introduced in DR Congo.

## Introduction

The nasopharynx is the niche for potential respiratory pathogens including *Streptococcus pneumoniae* and *Haemophilus influenzae* [1–3]. These are the main bacterial pathogens associated with acute lower respiratory infections (ALRIs) in children below 5 years of age [2, 3]. The introduction of conjugate vaccines for *S*. *pneumoniae* and *H*. *influenzae* type b over the past decades has significantly reduced the burden of bacterial infections in many countries [4]. The 13-valent pneumococcal conjugate vaccine (PCV13), which confers protection against 13 different pneumococcal serotypes was introduced in the routine child immunization program in 2013 in the Eastern DR Congo. A shift towards pneumococcal serotypes not included in the vaccine has been observed in many populations after the introduction of the PCVs [5].

Although most of the pathogens causing lung infections can be detected in the nasopharynx, the occurrence of these does not necessarily indicate disease [6]. In fact similar frequencies of pathogens have been found in healthy children as in children with ALRI [6, 7]. Nevertheless, studies of the nasopharyngeal microbiota of healthy children provide important information on the circulating respiratory viral and bacterial pathogens including distribution of pneumococcal serotypes and level of antibiotic resistance [8]. These factors are important for updating guidelines on the management of childhood ALRI. This is especially the case in countries and regions where there are little or no resources for microbiological testing of patients [8, 9].

To our knowledge, there have been no previous studies of the bacterial and viral loads in the upper respiratory tract of young Congolese children. In our recent analysis of the pneumococci colonizing the nasopharynx of children from the general population in DR Congo, we have found high rates of resistance to commonly used antibiotics [10]. In this study we applied molecular methods on the nasopharyngeal samples which had been obtained from the same cohort of Congolese children for the detection of a broad range of bacteria and viruses and for assessment of the distribution of pneumococcal serotypes with emphasis on the serotypes in PCV13.

## Methods

The study was approved by the Commission Institutionelle d’Ethique (CIE) of the Université Catholique de Bukavu (N/Ref: UCB/CIE/NC/22/2014) in accordance to existing ethical guidelines in D.R Congo and the Swedish regional ethical committee in Göteborg (N°: 504–16). The South-Kivu provincial Medical Doctor of Health in Bukavu was informed and approved the study (Ref: 065/CD/DPS-SK/2015). Informed oral and written consent was obtained from the accompanying parent or guardian of each child included in the study.

### Study population

From January 2014 to June 2015, 794 children aged from two to 60 months attending health centres in the Kivu province in the Eastern DR Congo for immunisation or growth monitoring were included in a culture-based study on pneumococcal carriage as previously described [10]. Nasopharyngeal samples collected from 375 of these children recruited at health centres in the city of Bukavu (Panzi, n = 80) or the surrounding rural area (Nyantende, Muku and Kaziba, n = 295) were made available for molecular analysis of microbial pathogens and pneumococcal serotypes. Written questionnaires relating to immunisation status and demographic factors were completed for the enrolled children either by trained final-year medical students or nurses in the presence of a paediatrician. For the 253 children enrolled in 2015, additional questionnaires relating to socio-economic conditions and previous illnesses were included. Basic physical examinations of all children were performed to monitor signs of respiratory tract infection. Weight and height were also measured.

### Specimen collection

A nasopharyngeal specimen was obtained from the participating children using a flocked swab transported in Amies Medium (ESwab^TM^, Copan Diagnostics Inc., Murrieta, CA). At each centre one qualified investigator collected the sample following a standardised procedure as previously described [10]. The samples were transported to the Clinical Laboratory at Panzi Hospital within two to six hours for subsequent pneumococcal culture [10]. The samples were then stored at -20°C prior to shipment to The Department of Infectious Diseases, University of Gothenburg, Gothenburg, Sweden. Here they were stored at -80°C until analysed (see below).

### Bacterial and viral nucleic acid detection

Nucleic acids extracted from 200 μL of the nasopharyngeal sample using a MagNA Pure LC instrument (Roche Diagnostics, Mannheim, Germany) and the Total Nucleic acid Isolation kit (Roche Diagnostic) were eluted in 100 μL elution buffer and were then stored at -20°C awaiting further analysis.

A previously described multiplex real-time PCR assay was used for detection of 15 different viruses (adenovirus, bocavirus, coronavirus 229E, HKU1, NL63 and OC43, enterovirus, influenza A and B, human metapneumovirus, parainfluenza 1–3, rhinovirus and respiratory syncytial virus (RSV)) and 5 different bacterial species (*Bordetella pertussis*, *Chlamydia pneumoniae*, *H*. *influenzae*, *Mycoplasma pneumoniae* and *S*. *pneumoniae*) [11]. A PCR Cycle threshold (Ct)-value of <35 was considered a positive result with values of Ct<30 indicating high levels of nucleic acids. Pneumococci were identified both by detection of the *lytA* gene included in the multiple pathogen panel, mentioned above and by occurrence of the *cpsA* gene included in the serotyping multiplex real-time PCR panel (see below). A sample was considered positive for pneumococci if one or both of these genes were detected. For analysis of pneumococci at high levels, either *lytA* or *cpsA*, or both, were detected at Ct levels below 30.

### Pneumococcal serotyping

A multiplex real-time PCR capable of detecting 40 different serotypes/serogroups including the 13 serotypes in PCV13 was performed according to a previously published protocol [12]. Each multiplex included the pneumococcal capsule-coding gene *cpsA* in order to verify the occurrence of pneumococcal DNA in the sample. In addition, two pUC57 plasmids containing PCR target amplicons for all serotypes were included in the panel as positive controls.

### Data management and statistical analysis

Descriptive analysis was performed using the SPSS package (version 24.0) for logistic regression to analyse the relationship between nucleic acid identification and socio-demographic or medical factors. Prevalence rates and the 95% confidence interval (CI) were calculated. Potential variables associated with identified bacteria or viruses were assessed by odds ratios (OR) with a 95% CI and were tested by univariable analysis with the Pearson’s chi-squared test or the Fisher’s exact test (if n < 5). Associations with p-values of *p* < 0.2 were re-analysed by multivariable analysis. A *p-*value of < 0.05 was considered statistically significant. The Mann-Whitney U-test was used for comparison of medians and the unpaired t-test for Ct-values in relation to socio-demographic factors. As classified by the Emergency Nutrition Assessment (ENA) software (version 2011), malnutrition was defined as either the weight for the subjects’ age or weight vs. height giving a Z score of ≤ -2 standard deviations.

## Results

### Detection of bacterial and viral nucleic acids

S. *pneumonia*e was the most common pathogen, detected by real-time PCR in 86% of the nasopharyngeal samples whereas *H*. *influenzae* was detected in 69% **(Table 1).** When employing a more stringent cut-off level (Ct <30), bacteria were found in 83% of the samples; S. *pneumonia*e was found in 77% of the cases and *H*. *influenzae* in 51% (**Table 1).** The most frequently detected virus was rhinovirus (49%) followed by enterovirus and parainfluenza virus in 22% and 17% of the samples, respectively. RSV was only found in 1% of the cases, and influenza virus was rarer still (**Table 1**). When only high levels of viral nucleic acids were considered (Ct-value of <30), rhinovirus was found in 30% of the cases and only a few samples were positive for enterovirus or any other respiratory virus **(Table 1).** Co-occurrence of bacteria and viruses was common; for 269 (72%) of the children at least one bacterium and one virus were detected when using a cut-off level of Ct<35, and 122 (33%) when a cut-off level of Ct<30 was used. Pneumococci together with any virus, both at high levels (Ct<30), were detected in 114 (30%) children.

**Table 1:** Detection of pathogens by real-time PCR using the cut-off levels Cycle threshold, Ct- value of <35 (all positive) and Ct<30 (only high levels), respectively, in nasopharyngeal secretions from 375 children at age 2–60 months attending health centres for scheduled routine immunisation or growth monitoring.

	*N (%) positive by real-time PCR*		
*Pathogens*	*High level Ct<30*	*Any level Ct<35*	*Ct-values in positive samples (median*, *range)*
***Streptococcus pneumoniae***	289 (77)	324 (86)	25.3 (18.0–34.7)
***Haemophilus influenzae***	190 (51)	257 (69)	27.0 (18.5–34.9)
***Bordetella pertussis***	4 (1)	11 (3)	30.8 (25.5–34.5)
***Chlamydia pneumoniae***	3 (1)	7 (2)	32.8 (24.8–34.8)
***Mycoplasma pneumoniae***	2 (0.5)	3 (1)	29.4 (25.1–33.4)
**Any bacteria**	313 (83)	349 (93)	-
**Rhinovirus**	113 (30)	182 (49)	29.1 (20.2–34.9)
**Enterovirus**	26 (7)	81 (22)	32.0 (19.2–34.9)
**Parainfluenza virus**	6 (2)	63 (17)	32.0 (19.4–34.9)
**Coronavirus**	7 (2)	20 (5)	30.9 (26.5–34.8)
**Bocavirus**	9 (2)	17 (5)	29.5 (15.8–34.7)
**Adenovirus**	11 (3)	14 (4)	26.9 (16.3–34.0)
**RSV**	2 (0.5)	4 (1)	28.7 (10.0–33.7)
**Human metapneumovirus**	4 (1)	7 (2)	29.2 (20.3–34.6)
**Influenza A virus**	0 (0)	1 (0.3)	34.5
**Any virus**	147 (39)	282 (75)	-
**Co-occurrence of any bacteria and any virus**	122 (33)	269 (72)	-

RSV = Respiratory syncytial virus

### Associations between pathogens and socio-demographic or medical factors

**Fig 1:**
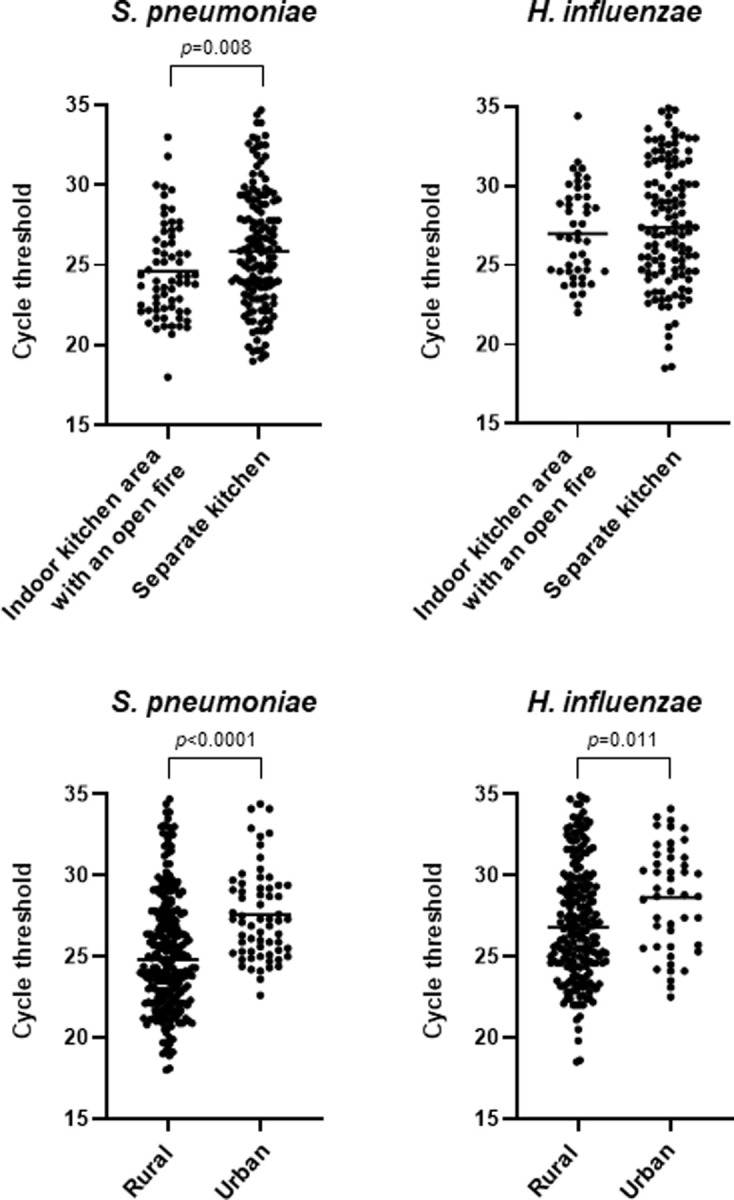
Socio-demographic factors in relation to pneumococci and H. influenzae detected by real-time PCR in nasopharyngeal secretions. PCR Cycle threshold (Ct) values <35 for the detection of the pathogens and means are shown (for pneumococci only detection of the LytA gene is shown). The lower Ct value, the higher amounts of nucleic acids are present in the sample. Indoor kitchen area with an open fire: Kitchen with an open fire located inside the dwelling being directly connected to the living room and/or the bedrooms. Separate kitchen: Kitchen which may located inside the dwelling but not having an open fire, or it may be located inside the dwelling there being no direct connection with the living room and/or bedrooms, or it may be located outside the dwelling.

A higher frequency of pneumococci at high levels (Ct<30) was observed in the children living in rural areas as compared to urban locations (80% versus 67%; OR 1.92; 95% CI 1.11–3.33; *p* = 0.019) and among children who lived in dwellings with an indoor kitchen area with an open fire, i.e. a dwelling with a cooking area with an open fire located inside the dwelling with the resultant smoke passing to the living room and/or the bedrooms as compared to those children living in a dwelling not having an indoor kitchen area with an open fire, or a kitchen not directly connected to the bedrooms or living room, or a kitchen located outside the dwelling (88% vs. 66%; OR 2.97; 95% CI 1.38–6.41; *p* = 0.005) (**Table 2**). The mean Ct-levels of detected pneumococci were also lower in children living in a dwelling with an indoor kitchen area with an open fire with the resultant exposure to indoor smoke from the cooking area as compared to those not living in this type of dwelling (**Fig 1**). Significantly lower Ct-values (i.e. a higher bacterial load) were also detected for both pneumococci and *H*. *influenzae* in children living in rural areas as compared to those living in the Bukavu urban areas (**Fig 1**). No significant associations were found between high levels of pneumococci and age, sex, number of people living in the house, parental smoking, number of siblings nor having animals in the house (**Table 2)**. Immunization with 2–3 doses of PCV13 was associated with slightly lower rates of pneumococci detected at high levels (Ct-value of <30) (OR 0.57; 95% CI 0.33–0.98; *p* = 0.045) (**Table 3**). Also, by using the cut-off level of Ct<35, a small reduction in the carriage of pneumococci could be detected between children who had received 2–3 doses of PCV13 as compared to those who had received none or just 1 dose (82% versus 91%; OR 0.49; 95% CI: 0.26–0.92, *p* = 0.026). There was no significant association between occurrence of any of the viruses detected at high levels (Ct-value of <30) and any of the socio-demographic or medical factors reported (S1 Table).

**Table 2:** Socio-demographic factors in relation to pneumococci detected at high levels by real-time PCR in nasopharyngeal secretions from 375 children at age 2–60 months attending health centres for scheduled routine immunisation or growth monitoring. Data are presented using the PCR cut-off level of Ct <30.

*Socio-demographic factors*		*Pneumococci detected / N (%)*	*OR (95% CI)*	*p-value*
***Sex***	***Girls***	152/199 (76)	0.89 (0.54–1.44)	0.63
	***Boys***	138/176 (75)	1.12 (0.69–1.82)	0.63
***Age in months***	***< 6***	65/88 (74)	1.00	
	***6–12***	79/103 (77)	1.16 (0.60–2.25)	0.65
	***> 12–24***	50/65 (77)	1.17 (0.55–2.49)	0.66
	***> 24–36***	51/62 (82)	1.64 (0.73–3.67)	0.22
	***> 36–60***	45/57 (79)	1.32 (0.59–2.93)	0.48
***Health centres***	***Panzi***	54/80 (67)	1.00	
	***Nyantende***	***89/98 (91)***	***4*.*28 (1*.*91–9*.*57)***	***0*.*0004***
	***Muku***	***86/105 (82)***	***2*.*17 (1*.*10–4*.*31)***	***0*.*025***
	***Kaziba***	61/92 (66)	0.94 (0.50–1.79)	0.86
***Location of residence***	***Urban***	54/80 (67)	1.00	
	***Rural***	***236/295 (80)***	***1*.*92 (1*.*11–3*.*33)***	***0*.*019***
***Number of people living in the dwelling***^1^	***1–5***	75/101 (74)	1.00	
	***> 5 - ≤ 10***	110/145 (76)	1.08 (0.60–1.95)	0.77
	***> 10***	5/7 (71)	0.86 (0.15–4.74)	0.86
***Siblings***^1^	***< 4***	160/214 (75)	1.00	
	***≥ 4***	30/39 (77)	1.12 (0.50–2.51)	0.77
***Having animals in the dwelling*** ^1^				
***Hen***		26/33 (79)	1.26 (0.52–3.08)	0.59
***Goat***		24/29 (83)	1.67 (0.61–4.59)	0.31
***Cow***		11/14 (79)	1.22 (0.33–4.55)	0.75
***Other animals***		37/48 (77)	1.14 (0.54–2.40)	0.72
***Partly breastfeeding (months)***^1^	***< 6***	73/103 (71)	1.00	
	***7–12***	93/118 (79)	1.52 (0.82–2.82)	1.17
	***> 12***	24/32 (75)	1.23 (0.49–3.05)	0.65
***Kitchen***^1^	***Separate kitchen***^2^	127/181 (70)	1.00	
	***Indoor kitchen area with an open fire***^3^	***63/72 (88)***	***2*.*9 7(1*.*38–6*.*41)***	***0*.*005***
***Most important fuel for cooking***^1^	***Electricity***	23/35(66)	1.00	
	***Wood***	72/93 (77)	1.78 (0.76–4.18)	0.18
	***Charcoal***	85/112 (76)	1.64 (0.72–3.73)	0.23
	***Combination (electricity + charcoal)***	10/13 (67)	1.73 (0.40–7.54)	0.45
***Parental tobacco smoking***^1^		10/11 (91)	3.44 (0.43–27.45)	0.24

^*1*^ These data were collected from 253 children

^2^ Separate Kitchen—Kitchen possibly located inside the dwelling but not having an open fire (for example electricity), or a kitchen located in the dwelling without a direct connection to the living room and/or bedrooms, or a kitchen located outside the dwelling.

^3^ Indoor kitchen area with an open fire—Kitchen area with an open fire located inside the dwelling with the resultant smoke passing to the living room and/or bedrooms

**Table 3:** Medical factors in relation to pneumococcal detection at high levels in nasopharyngeal secretions from 375 children aged 2–60 months attending health centres for scheduled routine immunisation or growth monitoring. Real-time PCR results with a cut-off level of Ct<30 are shown.

*Medical Factors*		*Pneumococci detected/N (%)*	*OR (95% CI)*	*p-Value*
***Immunisation Hib***^1^	***0 dose***	23/30 (77)	1.00	
	***1 dose***	45/59 (76)	0.97 (0.34–2.75)	0.96
	***2–3 doses***	222/286 (77)	1.05 (0.43–2.57)	0.90
***Immunisation PCV13***^2^	***0 doses***	142/176 (81)	1.00	
	***1 dose***	64/80 (80)	0.95 (0.49–1.85)	0.89
	***2–3 doses***	***84/119 (71)***	***0*.*57 (0*.*33–0*.*98)***	***0*.*045***
***Ongoing symptoms***^3^	***Fever***	17/19 (89)	2.99 (0.67–13.35)	0.14
	***Cough***	66/81 (81)	0.67 (0.41–1.09)	0.11
	***Runny nose***	18/21 (86)	2.09 (0.59–7.35)	0.24
	***Others symptoms***^4^	47/58 (81)	1.63 (0.78–3.39)	0.19
***History of disease***^3^	***Malaria***	26/34 (76)	1.08 (0.46–2.54)	0.84
	***Gastroenteritis***	23/34 (68)	0.65 (0.29–1.42)	0.28
	***Asthma***	10/12 (83)	1.69 (0.36–7.94)	0.50
	***Neonatal problems***	39/48 (81)	1.54 (0.70–3.40)	0.27
	***History of hospitalization***	43/61 (70)	0.73 (0.38–1.39)	0.34
	***Other diseases***^5^	21/25 (84)	1.83 (0.60–5.55)	0.28
	***History of antibiotic treatment (last month)***	33/45 (73)	0.89 (0.42–1.85)	0.76

^*1*^ *Hib*: *Haemophilus influenzae* type b vaccine

^*2*^ *PCV13*: 13-valent pneumococcal conjugate vaccine

^3^ These data were collected from 253 children

^4 ^Others symptoms: abdominal pain, skin rash, headache

^5^ Other diseases: injuries, meningitis, and post-surgery complications

### Pneumococcal serotypes/serogroups detected by real-time PCR

**Fig 2:**
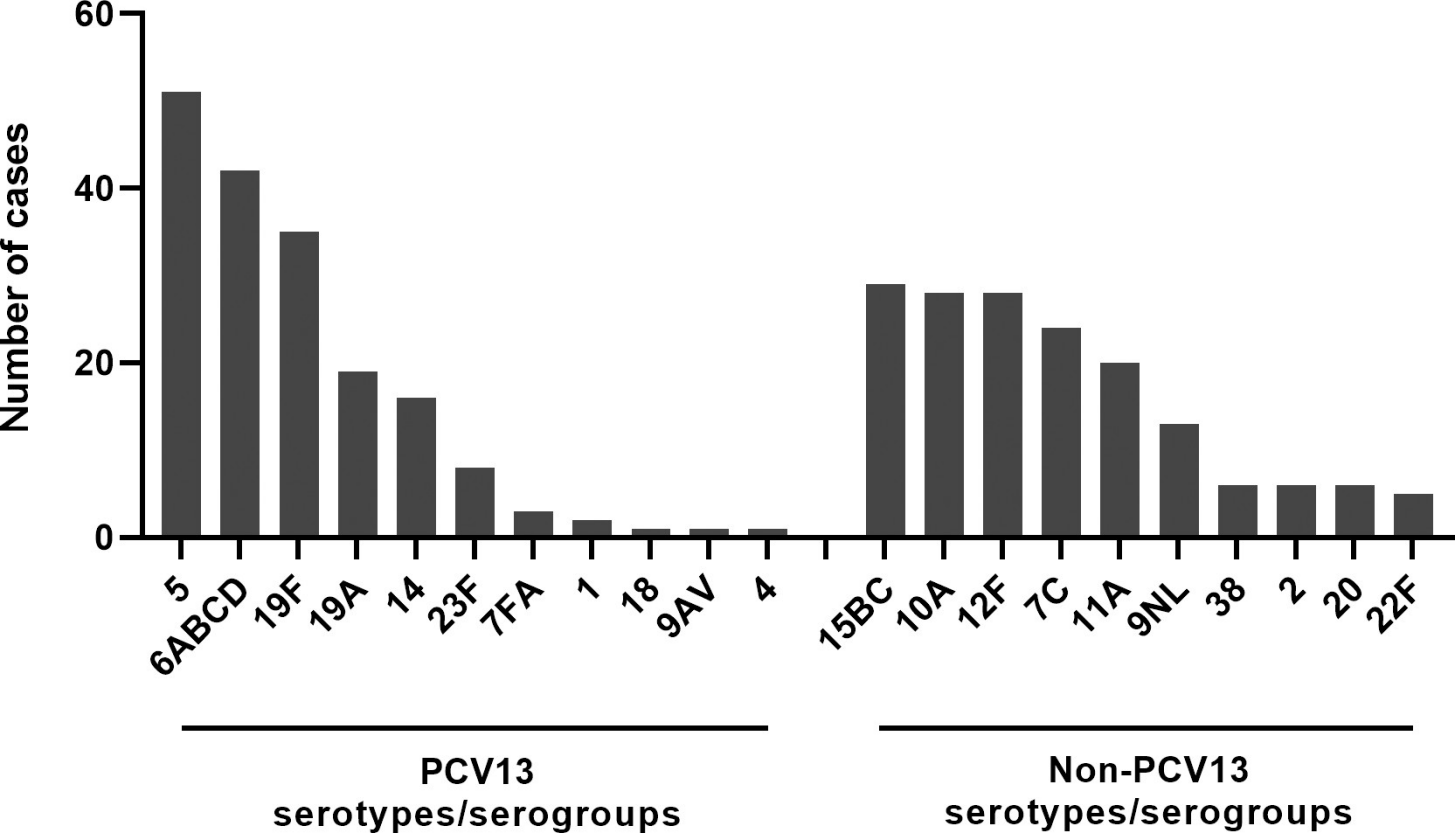
Identification of 344 pneumococcal serotypes in the nasopharyngeal secretions from 217 children out of 324 children positive for pneumococci by real-time PCR.

From the 324 nasopharyngeal samples that were positive for pneumococci, 217 (67%) were positive for at least one serotype/serogroup. However, our PCR panel could not identify any pneumococcal serotypes/serogroups in the remaining 107 (33%) nasopharyngeal samples in which pneumococci had been detected. One serotype/serogroup was detected in 127 samples, two serotypes in 61, three serotypes in 22, four serotypes in six samples and five serotypes in one sample. From all 344 serotypes/serogroups identified, 179 (52%) belonged to PCV13, while 165 (48%) were non-PCV13 serotypes/serogroups. Among the PCV13 serotypes/serogroups detected, serotype 5 was the most prevalent (15%) followed by serogroup 6 (12%) and serotype 19F (10%) (**Fig 2**). The most common non-PCV13 serotypes were 15BC, 10A and 12F (8% for all, **Fig 2**). In children from rural areas, serotype 5 was detected significantly more often than those from urban areas while serotype 12F was more prevalent in urban areas than rural areas **(Table 4).** PCV13 serotypes/serogroups were as common among children who had received 2–3 doses of PCV13 as they were in children who had received none or just 1 dose (48/96, 50% vs. 131/248, 53%). Sixty samples were positive for the *cpsA* gene which specifies the pneumococcal capsule but were negative for any serotype included in the panel. From all samples, 49 were negative for *cpsA* but were positive for *lytA*, indicating the occurrence of non-encapsulated non-typeable pneumococci.

**Table 4:** Pneumococcal serotypes/serogroups (n = 344) detected in nasopharyngeal secretions from 217 children aged 2–60 months living in rural or urban areas in the Eastern DR Congo.

	*Number of cases (%)*			
*Pneumococcal serotype/serogroup*	*Rural area n = 293*	*Urban area n = 51*	*OR (95% CI)*	*p-value*
***PCV13 serotypes/serogroups***				
***5***	***50 (17)***	***1 (2)***	***10*.*28 (1*.*38–76*.*22)***	***0*.*022***
***6ABCD***	36 (12)	6 (12)	1.05 (0.41–2.63)	0.91
***19F***	33 (11)	2 (4)	3.10 (0.72–13.38)	0.12
***19A***	15 (5)	4 (8)	0.63 (0.20–1.99)	0.43
***14***	13 (4)	3 6)	0.74 (0.20–2.70)	0.65
***23F***	7 (2)	1 (2)	1.22 (0.14–10.16)	0.85
***7FA***	3 (1)	0 (0)	-	-
***1***	2 (1)	0 (0)	-	-
***18***	1 (0.3)	0 (0)	-	-
***9AV***	1 (0.3)	0 (0)	-	-
***4***	1 (0.3)	0 (0)	-	-
***Non-PCV13 serotypes/serogroups***				
***15BC***	22 (8)	7 (14)	0.51 (0.20–1.26)	0.14
***10A***	21 (7)	7 (14)	0.48 (0.19–1.20)	0.12
***12F***	***20 (7)***	***8 (16)***	***0*.*39 (0*.*16–0*.*95)***	***0*.*038***
***7C***	20 (7)	4 (8)	0.86 (0.28–2.63)	0.79
***11A***	16 (5)	4 (8)	0.67 (0.21–2.11)	0.50
***9NL***	10 (3)	3 (6)	0.56 (0.15–2.12)	0.39
***38***	6 (2)	0 (0)	-	-
***2***	6 (2)	0 (0)	-	-
***20***	5 (2)	1 (2)	0.86 (0.99–7.58)	0.89
***22FA***	5 (2)	0 (0)	-	-

## Discussion

In the present study we assessed the prevalence of potential pathogenic bacteria and viruses in the nasopharynx of healthy children in the DR Congo. This is important for the future prevention and management of acute lower respiratory infections in the area. To our knowledge, there are no other published studies from the country using a broad approach for detection of respiratory pathogens in children.

The most common bacteria detected by PCR were pneumococci and *H*. *influenzae*; these were detected in 86% and 69% of the included children, respectively. A similar rate of pneumococci as detected by PCR from nasopharyngeal secretions of healthy children was reported from Zanzibar, Tanzania (83%)[7] while lower rates were reported for children from the general population in Thailand (58%) and from children attending day-care centres in the United Kingdom (65%) [13, 14]. The variation in detection rates between the different studies could be explained by differences in the sensitivity and cut-off levels of the PCR methods used for microbial detection [15]. The high pneumococcal detection rates reported in this and other studies may also be due to high numbers of local infection foci with bacterial transmissions between children in this area. At the time of this study, 68% of children had either not received PCV13 or only a single dose, which might contribute to the high detection rate. We found that immunisation with 2 or 3 doses of PCV13 was associated with lower prevalence of pneumococci thus corroborating earlier findings in Ethiopia [16] and the findings of our previous study [10] although this reduction was modest. A reducing effect of PCV13 immunisation on pneumococcal carriage has been reported and proven in several studies [17]. However, carriage of both PCV13 and non-PCV13 serotypes are common among Sub-Saharan children both before and after introduction of the conjugate vaccines [18–21] and we did not report any differences in serotype distribution between vaccinated and non-vaccinated children.

The carriage rate of pneumococci was high even when only high levels of nucleic acids were considered (77%, Ct value <30). We previously assessed pneumococcal carriage by culture from the same cohort of patients. This indicated a rather low prevalence (20%) as compared to other studies performed in the post-PCV13 era in Sub-Saharan Africa, in particular Malawi, the Gambia, South Africa, Kenya, Ethiopia and Mozambique [19–24]. The results in this study indicate that culture methodology limitations could account for the relatively low pneumococcal prevalence in Congolese children. The use of molecular methods may contribute further valuable information concerning circulating pathogens within the population.

We found associations between high nucleic acid levels for both pneumococci and *H*. *influenzae* in the nasopharynx of children living in rural areas. By using detection by culture we previously showed a similar association for pneumococci [10]. The same observation was also described in Laos among children 1–2 years of age [25]. As reported in our previous culture-based study, we can also confirm an association between pneumococcal detection at high levels and dwellings having an indoor kitchen area with an open cooking fire [10]. It remains to be investigated whether the higher load of pneumococci present in these children is associated with exposure to toxic smoke resulting from the presence of an indoor cooking area with an open fire.

The most frequently detected virus was rhinovirus (49%), followed by enterovirus (22%) and parainfluenza virus (17%). Rhinovirus was the most frequently detected virus in the healthy control group of a multi-centre childhood pneumonia study in eight low- and middle-income countries in Africa, Asia and South America [26]. Rhinovirus followed by enterovirus (26% and 12%, respectively) was also reported as being the most prevalent virus detected in the nasopharynx of children attending day-care centres in Norway [27]. However, these viruses were less prevalent than in the Congolese children. Many factors may influence regional differences in viral detection rates such as seasonal variations, outbreaks, environmental factors and genetics [28]. Rhinovirus was found to be equally common in pneumonia cases as in control children in the Pneumonia Etiology Research and Child Health (PERCH) multi-center study performed in seven countries in Africa and Asia [6]. Also, in children from the Soweto township of Johannesburg in South Africa the prevalence of rhinovirus was similar among pneumonia cases and controls (23% versus 22%) with a much lower prevalence in the control group as compared to the findings of our study [29]. In Russia, rhinovirus was more frequently detected in healthy controls than in children with community-acquired pneumonia; the same was found in Tanzania for control children as compared to those with acute febrile illness[7, 30]. A study from USA of under-fives reported an equal prevalence of coronaviruses (other than SARS-CoV-2) in hospitalized cases with fever and/or acute respiratory illness compared to controls (8% versus 7%) which was similar to our observed prevalence of 5% [31]. Our data confirm previous observations that potential pathogens are abundant in healthy children. Consequently, the detection of pathogenic bacteria or viruses in the upper respiratory tract of sick children should be interpreted with caution. Several studies have reported RSV to be among the most frequently detected viruses in the nasopharynx of children with pneumonia; however, a significantly lower frequency was reported in the controls [6, 26, 30]. In the present study, only a few children had a positive detection of RSV in the nasopharynx.

Co-occurrence of bacteria and viruses was common in our study, as measured by either the higher (Ct-value <35) or the lower (Ct-value <30) detection level with rates of 72% and 33%, respectively. A similar rate (76%) of bacterial and viral co-occurrence was reported among the control children from the general population in the PERCH multi-center pneumonia study [6, 29]. Variable rates of multiple pathogens have been described in Gambian infants (94%), in healthy Tanzanian children (83%) and in healthy children aged from 6 weeks to 24 months in the Netherlands (38%) [32–34].

In the present study serotype 19F was among the most prevalent PCV13 serotypes in the detected pneumococci. This was similar to findings from Uganda [35] and our previous culture-based study [10]. However, the highest prevalence of serotype 5 recorded by PCR directly on the nasopharyngeal samples was different to the serotype prevalences identified by cultural isolation from the same cohort of children [10]. This suggests molecular methods to be a valuable complement to detection by culture for assessment of circulating pneumococcal serotypes. While serotype 19A was fairly prevalent, serotype 3 was not detected at all. Both these serotypes, together with 6A, were added in PCV13; however, these were not present in the 7-valent nor the 10-valent pneumococcal conjugate vaccines. Despite the introduction of PCV13 immunisation serotype 3 still causes invasive pneumococcal disease in many countries. Several studies indicate that this may be a consequence of the poor protection of PCV13 against serotype 3 colonization [36]. It has also been reported that serotype 19A pneumococci were still at 5% among children in New York five years after PCV13 introduction. However, there had been a significant drop in overall pneumococcal incidence since the pre-vaccine era [5].

At 48%, the prevalence of non-PCV13 serotypes/serogroups among the Congolese children was high. However, the PCR method cannot differentiate all the serotypes in serogroup 6, of which only 6A and 6B are included in PCV13. Similarly, for serogroup 9, only 9V is included in PCV13, thus implying that the prevalence of non-PCV13 serotypes may be higher than indicated in this study. Moreover, 107 samples which were positive for pneumococci were negative in our serotype PCR assay. This method has been designed primarily to detect vaccine-containing serotypes. Therefore, the prevalence of non-PCV13 serotypes may be high. Forty-nine of our samples were negative for the pneumococcal capsule gene *cpsA* but were positive for the pneumococcal autolysin *lytA* gene thus suggesting occurrence of non-encapsulated non-typeable pneumococci. However, no analyses have been performed to confirm this observation. A high prevalence of non-PCV13 serotypes has previously been described in the Gambia [19], Malawi [37] and Tanzania [38] probably due to serotype replacement following PCV introduction as observed in many countries [19, 39, 40]. The geographic disparity of pneumococcal carriage around the world may contribute to this phenomenon [41, 42].

Using either detection by culture or molecular methods serotypes 10A and 15BC were among the most prevalent non-PCV13 serotypes in the Congolese children [10]. The non-PCV13 serotypes 15BC followed by 10A, 21 and 16F were also the most prevalent serotypes in South-African infants after introduction of PCV13 vaccination in South Africa [20]. Differences in serotype distribution might be explained by the geographic heterogeneity of circulating serotypes and may be attributable to environmental factors such as crowding or genetic factors [42].

In 90 samples we were able to detect more than one serotype indicating carriage of more than one pneumococcal strain. However, the clinical significance of this remains unclear. The interplay between commensals and potential pathogens on the upper respiratory mucosa in health and disease is complex and thus warrants further studies on the nasopharyngeal microbiome.

## Conclusions

We report a high prevalence of both bacteria and viruses in the nasopharynx of children from the general population in DR Congo. The bacteria included *S*. *pneumoniae* and *H*. *influenzae*. The viruses were predominantly rhinovirus and enterovirus with a low level of RSV. Both living in a rural area and having an indoor kitchen area with an open fire were associated with a higher pneumococcal load while PCV13 vaccination was associated with lower rates of pneumococcal detection. Our results show a predominance of non-PCV13 serotypes among children in DR Congo.

## Supporting information

S1 Table (DOC)

## References

[1] Bortz K, Neumann RP. Nose, throat microbiome hints at source of LRTIs in children. Infectious Diseases in Children. 2019;32(5):20–20.

[2] Dubourg G, Edouard S, Raoult D. Relationship between nasopharyngeal microbiota and patient’s susceptibility to viral infection. Expert Review of Anti-infective Therapy. 2019;17(6):437–47. 10.1080/14787210.2019.1621168 31106653

[3] Rudan I, Boschi-Pinto C, Biloglav Z, Mulholland K, Campbell H. Epidemiology and etiology of childhood pneumonia. Bulletin of the world health organization. 2008;86:408–16B. 10.2471/blt.07.048769 18545744 PMC2647437

[4] Wahl B, O’Brien K, Greenbaum A, Liu L, Chu Y, Majumder A, et al Global, regional, and national burden of Streptococcus pneumoniae and Haemophilus influenzae type b disease in children in the era of conjugate vaccines: updated estimates from 2000–2015. Lancet Glob Health. 2018;6:e744–57. 10.1016/S2214-109X(18)30247-X 29903376 PMC6005122

[5] Kaur R, Casey JR, Pichichero ME. Emerging Streptococcus pneumoniae Strains Colonizing the Nasopharynx in Children after 13-Valent (PCV13) Pneumococcal Conjugate Vaccination in Comparison to the 7-Valent (PCV7) Era, 2006–2015. The Pediatric infectious disease journal. 2016;35(8):901 10.1097/INF.0000000000001206 27420806 PMC4948952

[6] O'Brien KL, Baggett HC, Brooks WA, Feikin DR, Hammitt LL, Higdon MM, et al Causes of severe pneumonia requiring hospital admission in children without HIV infection from Africa and Asia: the PERCH multi-country case-control study. The Lancet. 2019;394(10200):757–79. 10.1016/S0140-6736(19)30721-4 31257127 PMC6727070

[7] Elfving K, Shakely D, Andersson M, Baltzell K, Ali AS, Bachelard M, et al Acute uncomplicated febrile illness in children aged 2–59 months in Zanzibar–Aetiologies, antibiotic treatment and outcome. PloS one. 2016;11(1). 10.1371/journal.pone.0146054 26821179 PMC4731140

[8] Man WH, van Houten MA, Mérelle ME, Vlieger AM, Chu MLJ, Jansen NJ, et al Bacterial and viral respiratory tract microbiota and host characteristics in children with lower respiratory tract infections: a matched case-control study. The Lancet Respiratory Medicine. 2019 10.1016/S2213-2600(18)30449-1 30885620 PMC7172745

[9] Goga AE, Muhe LM, Forsyth K, Chopra M, Aboubaker S, Martines J, et al Results of a multi-country exploratory survey of approaches and methods for IMCI case management training. Health Research Policy and Systems. 2009;7(1):18 10.1186/1478-4505-7-18 19615080 PMC2723104

[10] Birindwa AM, Emgård M, Nordén R, Samuelsson E, Geravandi S, Gonzales-Siles L, et al High rate of antibiotic resistance among pneumococci carried by healthy children in the eastern part of the Democratic Republic of the Congo. BMC pediatrics. 2018;18(1):361 10.1186/s12887-018-1332-3 30453916 PMC6241069

[11] Andersson ME, Olofsson S, Lindh M. Comparison of the FilmArray assay and in-house real-time PCR for detection of respiratory infection. Scandinavian journal of infectious diseases. 2014;46(12):897–901. 10.3109/00365548.2014.951681 25288382

[12] Gonzales-Siles L, Salvà-Serra F, Degerman A, Nordén R, Lindh M, Skovbjerg S, et al Identification and capsular serotype sequetyping of Streptococcus pneumoniae strains. Journal of Medical Microbiology. 2019;68(8):1173–88. 10.1099/jmm.0.001022 31268417

[13] Piralam B, Prosperi C, Thamthitiwat S, Bunthi C, Sawatwong P, Sangwichian O, et al Pneumococcal colonization prevalence and density among Thai children with severe pneumonia and community controls. PloS one. 2020;15(4):e0232151 10.1371/journal.pone.0232151 32348330 PMC7190126

[14] Thors V, Christensen H, Morales-Aza B, Oliver E, Sikora P, Vipond I, et al High-density Bacterial Nasal Carriage in Children Is Transient and Associated With Respiratory Viral Infections—Implications for Transmission Dynamics. The Pediatric infectious disease journal. 2019;38(5):533–8. 10.1097/INF.0000000000002256 30985547

[15] Espy M, Uhl J, Sloan L, Buckwalter S, Jones M, Vetter E, et al Real-time PCR in clinical microbiology: applications for routine laboratory testing. Clinical microbiology reviews. 2006;19(1):165–256. 10.1128/CMR.19.1.165-256.2006 16418529 PMC1360278

[16] Negash AA, Asrat D, Abebe W, Hailemariam T, Gebre M, Verhaegen J, et al, editors. Pneumococcal Carriage, Serotype Distribution, and Risk Factors in Children With Community-Acquired Pneumonia, 5 Years After Introduction of the 10-Valent Pneumococcal Conjugate Vaccine in Ethiopia Open forum infectious diseases; 2019: Oxford University Press US. 10.1093/ofid/ofz259 PMC6592415 31263735

[17] Roca A, Bojang A, Bottomley C, Gladstone RA, Adetifa JU, Egere U, et al Effect on nasopharyngeal pneumococcal carriage of replacing PCV7 with PCV13 in the Expanded Programme of Immunization in The Gambia. Vaccine. 2015;33(51):7144–51. 10.1016/j.vaccine.2015.11.012 26592141 PMC5352730

[18] Usuf E, Bottomley C, Adegbola RA, Hall A. Pneumococcal carriage in sub-Saharan Africa—a systematic review. PloS one. 2014;9(1). 10.1371/journal.pone.0085001 24465464 PMC3896352

[19] Usuf E, Bottomley C, Bojang E, Cox I, Bojang A, Gladstone R, et al Persistence of nasopharyngeal pneumococcal vaccine serotypes and increase of nonvaccine serotypes among vaccinated infants and their mothers 5 years after introduction of pneumococcal conjugate vaccine 13 in The Gambia. Clinical Infectious Diseases. 2019;68(9):1512–21. 10.1093/cid/ciy726 30165376 PMC6481996

[20] Dube FS, Ramjith J, Gardner-Lubbe S, Nduru P, Robberts FL, Wolter N, et al Longitudinal characterization of nasopharyngeal colonization with Streptococcus pneumoniae in a South African birth cohort post 13-valent pneumococcal conjugate vaccine implementation. Scientific reports. 2018;8(1):1–9. 10.1038/s41598-017-17765-5 30131607 PMC6104038

[21] Swarthout TD, Fronterre C, Lourenço J, Obolski U, Gori A, Bar-Zeev N, et al High residual carriage of vaccine-serotype Streptococcus pneumoniae after introduction of pneumococcal conjugate vaccine in Malawi. Nature communications. 2020;11(1):1–12. 10.1038/s41467-019-13993-7 32376860 PMC7203201

[22] Heath CJ, Nayakwadi-Singer M, King CH, Malhotra I, Mutuku F, Mukoko D, et al Nasopharyngeal carriage of Streptococcus pneumoniae in children in coastal Kenya. The American journal of tropical medicine and hygiene. 2018;98(4):1046–50. 10.4269/ajtmh.16-0813 29488456 PMC5928806

[23] Abaye G, Fekadu H, Haji K, Alemu D, Anjulo AA, Yadate DT. Prevalence and risk factors of pneumococcal nasopharyngeal carriage in healthy children attending kindergarten, in district of Arsi Zone, South East, Ethiopia. BMC research notes. 2019;12(1):253 10.1186/s13104-019-4283-3 31064380 PMC6505268

[24] Adebanjo T, Lessa FC, Mucavele H, Moiane B, Chauque A, Pimenta F, et al Pneumococcal carriage and serotype distribution among children with and without pneumonia in Mozambique, 2014–2016. PloS one. 2018;13(6). 10.1371/journal.pone.0199363 PMC6019677 29944695

[25] Dunne EM, Choummanivong M, Neal EF, Stanhope K, Nguyen CD, Xeuatvongsa A, et al Factors associated with pneumococcal carriage and density in infants and young children in Laos PDR. PloS one. 2019;14(10). 10.1371/journal.pone.0224392 31661527 PMC6818791

[26] Bénet T, Sánchez Picot V, Messaoudi M, Chou M, Eap T, Wang J, et al Microorganisms associated with pneumonia in children< 5 years of age in developing and emerging countries: the GABRIEL pneumonia multicenter, prospective, case-control study. Clinical Infectious Diseases. 2017;65(4):604–12. 10.1093/cid/cix378 28605562 PMC7108107

[27] Moe N, Pedersen B, Nordbø SA, Skanke LH, Krokstad S, Smyrnaios A, et al Respiratory virus detection and clinical diagnosis in children attending day care. PLoS One. 2016;11(7). 10.1371/journal.pone.0159196 27433803 PMC4951077

[28] Tahamtan A, Samadizadeh S, Rastegar M, Nakstad B, Salimi V. Respiratory syncytial virus infection: why does disease severity vary among individuals? Expert Review of Respiratory Medicine. 2020;(just-accepted). 10.1080/17476348.2020.1724095 31995408

[29] Baillie VL, Moore DP, Mathunjwa A, Morailane P, Simões EA, Madhi SA. A prospective case-control study on the association of Rhinovirus nasopharyngeal viral load and viremia in South African children hospitalized with severe pneumonia. Journal of Clinical Virology. 2020;125:104288 10.1016/j.jcv.2020.104288 32092643 PMC7086148

[30] Spichak TV, Yatsyshina SB, Кatosova LК, Кim SS, Korppi MO. Is the role of rhinoviruses as causative agents of pediatric community-acquired pneumonia over-estimated? European journal of pediatrics. 2016;175(12):1951–8. 10.1007/s00431-016-2791-x 27714467 PMC7087148

[31] Prill MM, Iwane MK, Edwards KM, Williams JV, Weinberg GA, Staat MA, et al Human coronavirus in young children hospitalized for acute respiratory illness and asymptomatic controls. The Pediatric infectious disease journal. 2012;31(3):235 10.1097/INF.0b013e31823e07fe 22094637 PMC3288315

[32] Kwambana BA, Barer MR, Bottomley C, Adegbola RA, Antonio M. Early acquisition and high nasopharyngeal co-colonisation by Streptococcus pneumoniae and three respiratory pathogens amongst Gambian new-borns and infants. BMC infectious diseases. 2011;11(1):175 10.1186/1471-2334-11-175 21689403 PMC3129300

[33] van den Bergh MR, Biesbroek G, Rossen JW, de Steenhuijsen Piters WA, Bosch AA, van Gils EJ, et al Associations between pathogens in the upper respiratory tract of young children: interplay between viruses and bacteria. PloS one. 2012;7(10). 10.1371/journal.pone.0047711 23082199 PMC3474735

[34] Ngocho JS, Minja L, van der Gaast–de Jongh CE, Rahamat-Langendoen JC, Langereis JD, Mmbaga BT, et al Viral-bacterial (co-)occurrence in the upper airways and the risk of childhood pneumonia in resource-limited settings. Journal of Infection. 2020 10.1016/j.jinf.2020.06.013 32533999 PMC7392802

[35] Lindstrand A, Kalyango J, Alfven T, Darenberg J, Kadobera D, Bwanga F, et al Pneumococcal carriage in children under five years in Uganda-will present pneumococcal conjugate vaccines be appropriate? PloS one. 2016;11(11). 10.1371/journal.pone.0166018 27829063 PMC5102345

[36] Principi N, Di Cara G, Bizzarri I, Isidori C, Borgia P, Mignini C, et al Prevention of invasive pneumococcal disease: problems emerged after some years of the 13-valent pneumococcal conjugate vaccine use. Current Infectious Disease Reports. 2018;20(1):1 10.1007/s11908-018-0607-z 29368250

[37] Heinsbroek E, Tafatatha T, Phiri A, Swarthout TD, Alaerts M, Crampin AC, et al Pneumococcal carriage in households in Karonga District, Malawi, before and after introduction of 13-valent pneumococcal conjugate vaccination. Vaccine. 2018;36(48):7369–76. 10.1016/j.vaccine.2018.10.021 30352744 PMC6238076

[38] Emgård M, Msuya SE, Nyombi BM, Mosha D, Gonzales-Siles L, Nordén R, et al Carriage of penicillin-non-susceptible pneumococci among children in northern Tanzania in the 13-valent pneumococcal vaccine era. International Journal of Infectious Diseases. 2019;81:156–66. 10.1016/j.ijid.2019.01.035 30685588

[39] González-Díaz A, Càmara J, Ercibengoa M, Cercenado E, Larrosa N, Quesada M, et al Emerging non-13-valent pneumococcal conjugate vaccine (PCV13) serotypes causing adult invasive pneumococcal disease in the late-PCV13 period in Spain. Clinical Microbiology and Infection. 2019 10.1016/j.cmi.2019.10.034 31756452

[40] Esposito S, Principi N. Impacts of the 13-valent pneumococcal conjugate vaccine in children. Journal of immunology research. 2015;2015 10.1155/2015/591580 26351648 PMC4553318

[41] Hill PC, Akisanya A, Sankareh K, Cheung YB, Saaka M, Lahai G, et al Nasopharyngeal carriage of Streptococcus pneumoniae in Gambian villagers. Clinical infectious diseases. 2006;43(6):673–9. 10.1086/506941 16912937

[42] Dananché C, Paranhos-Baccalà G, Messaoudi M, Sylla M, Awasthi S, Bavdekar A, et al Serotypes of Streptococcus pneumoniae in Children Aged< 5 Years Hospitalized With or Without Pneumonia in Developing and Emerging Countries: A Descriptive, Multicenter Study. Clinical Infectious Diseases. 2020;70(5):875–83. 10.1093/cid/ciz277 31556939

