# Supplementary material for: High bacterial and viral load in the upper respiratory tract of children in the Democratic Republic of the Congo
Source: PLoS One. 2020 Oct 29;15(10):e0240922. doi: 10.1371/journal.pone.0240922 (PMC7595347; doi:10.1371/journal.pone.0240922)
Supplement: S1 Table — (DOC) [file pone.0240922.s001.doc]

*S1 Table: This shows the socio-demographic factors associated with viruses detected in the nasopharyngeal secretions of 375 children. These were aged from two to 60 months and were attending health centers for scheduled routine immunisation. Detection was recorded via real-time PCR with a Ct-Cycle threshold of Ct<30*

| ***Socio-demographic or medical factors*** | | ***N (%)*** | ***Any viruses detected N (%)*** | ***OR (95% CI)*** | ***p-value*** |
| --- | --- | --- | --- | --- | --- |
| ***Sex*** | ***Girls*** | 199 (53) | 88/199 (44) | 1.42 (0.93-2.15) | 0.09 |
|  | ***Boys*** | 176 (47) | 63/176 (36) | 0.70 (0.46-1.06) | 0.09 |
| ***Age in months*** | ***< 6*** | 88 (24) | 32/88 (36) | 1.00 |  |
|  | ***6 – 12*** | 103 (27) | 44/103 (43) | 1.30 (0.72-2.34) | 0.37 |
|  | ***> 12 – 24*** | 65 (17) | 22/65 (34) | 0.89 (0.45-1.75) | 0.74 |
|  | ***> 24 – 36*** | 62 (17) | 27/62 (44) | 1.35 (0.69-2.62) | 0.37 |
|  | ***> 36 – 60*** | 57 (15) | 26/57 (46) | 1.46 (0.74-2.89) | 0.26 |
| ***Health centres*** | ***Panzi*** | 80 (21) | 35/80 (44) | 1.00 |  |
|  | ***Nyantnende*** | 98 (26) | 46/98 (47) | 1.13 (0.62-2.05) | 0.67 |
|  | ***Muku*** | ***105 (28)*** | ***26/105 (25)*** | ***0.42 (0.22-0.79)*** | ***0.007*** |
|  | ***Kaziba*** | 92 (25) | 44/92 (48) | 1.17 (0.64-2.15) | 0.59 |
| ***Location of residence*** | ***Urban*** | 80 (21) | 35/80 (44) | 1.00 |  |
|  | ***Rural*** | 295 (79) | 116/295 (39) | 0.83 (0.50-1.37) | 0.47 |
| ***Number of people living in the dwelling^1^*** | ***1 - 5*** | 101 (40) | 33/101 (33) | 1.00 |  |
|  | ***> 5 - < 10*** | 145 (57) | 55/145 (38) | 1.25 (0.73-2.14) | 0.39 |
|  | ***> 10*** | 7 (3) | 3/7 (43) | 1.54 (0.32-7.30) | 0.58 |
| ***Siblings^1^*** | ***< 4*** | 214 (85) | 78/214 (36) | 1.00 |  |
|  | ***≥ 4*** | 39 (15) | 13/39 (33) | 0.87 (0.42-1.79) | 0.70 |
| ***Having animals in the dwelling^1^*** | ***Hen*** | 33 (13) | 14/33 (42) | 1.36 (0.65-2.87) | 0.40 |
|  | ***Goat*** | 29 (11) | 8/29 (28) | 0.64 (0.27-1.52) | 0.32 |
|  | ***Cow*** | 14 (6) | 6/14 (43) | 1.35 (0.45-4.04) | 0.55 |
|  | ***Other animals*** | 48 (19) | 16/48 (33) | 0.86 (0.44-1.68) | 0.67 |
| ***Partly breastfeeding (months)^1^*** | ***< 6*** | 103 (41) | 38/103 (37) | 1.00 |  |
|  | ***7-12*** | 118 (46) | 43/118 (36) | 0.98 (0.56-1.69) | 0.94 |
|  | ***> 12*** | 32 (13) | 10/32 (31) | 0.81 (0.34-1.91) | 0.63 |
| ***Kitchen^1^*** | ***Separate kitchen****^2^* | 181 (72) | 60/181(33) | 1.00 |  |
|  | ***Indoor kitchen area with an open fire****^3^* | 72 (28) | 31/72 (43) | 1.52 (0.87-2.66) | 0.13 |
| ***Most important fuel for cooking^1^*** | ***Electricity*** | 35 (14) | 16/35 (46) | 1.00 |  |
|  | ***Wood*** | 93 (37) | 36/93 (39) | 0.75 (0.34-1.64) | 0.47 |
|  | ***Charcoal*** | 112 (44) | 36/112 (32) | 0.56 (0.25-1.22) | 0.14 |
|  | ***Combination (electricity + charcoal)*** | 13 (5) | 3/13 (23) | 0.35 (0.08-1.52) | 0.16 |
| ***Parental tobacco smoking^1^*** |  | 11 (4) | 5/11 (45) | 1.51 (0.44-5.09) | 0.50 |
| ***Ongoing symptoms^1^*** | ***Fever*** | 19 (8) | 9/19 (47) | 1.66 (0.65-4.26) | 0.28 |
|  | ***Cough*** | 81 (32) | 29/81 (36) | 0.98 (0.57-1.71) | 0.96 |
|  | ***Runny nose*** | 21 (8) | 11/21 (52) | 2.09 (0.85-5.13) | 0.10 |
|  | ***Others symptoms****^4^* | 58 (23) | 24/58 (41) | 1.25 (0.68-2.30) | 0.45 |
| ***Past history of diseases^1^*** | ***Malaria*** | 34 (13) | 17/34 (50) | 1.95 (0.94-4.05) | 0.07 |
|  | ***Gastroenteritis*** | 34 (13) | 10/34 (29) | 0.70 (0.32-1.55) | 0.39 |
|  | ***Asthma*** | 12 (5) | 5/12 (42) | 1.28 (0.39-4.17) | 0.67 |
|  | ***Neonatal problems*** | 48 (19) | 20/48 (42) | 1.34 (0.70-2.56) | 0.36 |
|  | ***Prior hospitalization*** | 61 (24) | 24/61 (39) | 1.21 (0.66-2.19) | 0.52 |
|  | ***Other diseases****^5^* | 25 (10) | 8/25 (32) | 0.82 (0.34-1.98) | 0.66 |
| ***Immunisation Hib^6^*** | ***0 dose*** | 30 (8) | 15/30 (50) | 1.00 |  |
|  | ***1 dose*** | 59 (16) | 22/59 (37) | 0.59 (0.24-1.44) | 0.25 |
|  | ***2-3 doses*** | 286 (76) | 114/286 (40) | 0.66 (0.31-1.40) | 0.28 |
| ***Immunisation PCV13^7^*** | ***0 doses*** | 176 (47) | 76/176 (43) | 1.00 |  |
|  | ***1 dose*** | 80 (21) | 31/80 (39) | 0.83 (0.48-1.42) | 0.50 |
|  | ***2-3 doses*** | 119 (32) | 44/119 (37) | 0.77 (0.47-1.24) | 0.28 |

*^1^ These data were collected from 253 children*

*^2^ Indoor kitchen area with an open fire - Kitchen with an open fire located inside the dwelling with the resultant smoke passing to the living room and/or the bedrooms.*

*^3^ Separate Kitchen - Kitchen which may be located inside the dwelling but not having an open fire (eg. cooking with electricity) or it may be located inside the dwelling there being no direct connection with the living room and/or bedrooms, or it may be located outside the dwelling.*

*^4^ Others symptoms: abdominal pain, skin rash, headache*

*^5^ Other diseases: injuries, meningitis, and post-surgery complications*

*^6^ Hib: Haemophilus influenzae type b vaccine*

*^7^ PCV13: 13-valent pneumococcal conjugate vaccine*
